# Experiences of Nurses Caring for Patients With COVID‐19: A Descriptive Phenomenological Study

**DOI:** 10.1002/nop2.70601

**Published:** 2026-05-19

**Authors:** Jerry Armah, Joana Kyei‐Dompim, Edward Appiah Boateng, Kwaku Ankama Obour, Elizabeth Otu Inok, Selase Ewura Ama Obiri, Faithful Adwoa Annobil, Douglas Gyamfi, Abigail KUSI Amponsah

**Affiliations:** ^1^ Department of Nursing Kwame Nkrumah University of Science and Technology Kumasi Ghana; ^2^ Department of Nursing Science University of Turku Turku Finland

**Keywords:** COVID‐19 patients, experiences, Ghana, nursing care, pandemic, Sub‐Saharan Africa

## Abstract

**Aim:**

The current study aimed to explore the lived experiences of nurses providing care to patients with COVID‐19.

**Design:**

A descriptive phenomenological approach.

**Methods:**

The study was conducted among 9 purposively sampled nurses who worked in the COVID‐19 isolation centers of Kumasi South Hospital and Manhyia District Hospital, Kumasi. With the assistance of a semi‐structured interview guide and participants' consent, their lived experiences on caring for patients with COVID‐19 were audio‐recorded. The data were transcribed verbatim and inductively analysed into themes which described their experiences. The data were collected between the period of August and September 2021.

**Results:**

Participants comprised six females and three males; their ages ranged from 24 to 34 years. Participants had worked for 2–11 years in the profession and 4–15 months in the isolation unit. Three themes emerged from the data: ‘care context,’ ‘covid effects,’ and ‘coping strategies.’ ‘Care context’ referred to the working conditions in which the nurses provided care for the isolated patients. ‘Covid effects’ described the physical, psychological, and socio‐economic effects of the pandemic on the nurses and care provision. Participants also narrated their ‘coping strategies’ which were used in managing the care‐giving challenges posed by the pandemic.

**Patient Contribution:**

The study calls for the prioritisation of the well‐being and support of nurses working in isolation units, ensuring adequate resources, training, and protective measures to maintain their physical and psychological health. This will directly impact the care given to patients in such isolation units currently and in the future.

## 1 Introduction

The novel coronavirus (COVID‐19) pandemic has spread rapidly throughout the planet. Research shows that it originated from Wuhan province of China. It is highly contagious and has spread to all continents of the planet with a total number of 404,910,528 cases as at 14th February, 2022 (World Health Organization 2022). COVID‐19 has affected every country on the planet, and Africa is no exception. Africa was predicted to be the most vulnerable continent, with COVID‐19's spread having the greatest impact due to the continent's weak health system and immunocompromised population as a result of HIV/AIDS, anaemia, malaria, poor economic conditions, and other factors (Moore et al. 2017). The continent's first case of COVID‐19 was confirmed in Egypt on February 14, 2020, and the first case from Sub‐Saharan Africa was reported in Nigeria on February 27, 2020. The majority of COVID‐19 cases in Africa have been imported from Europe and the United States, rather than from China, where the virus originated (Lone and Ahmad 2020). Ghana recorded its first 2 cases of COVID‐19 on 12th March 2020. Both cases were imported with one from Turkey and the other from Norway (Ghana Health Service 2022).

## 2 Background

COVID‐19 has wreaked havoc on health‐care systems around the world, as elective medical services have been cancelled, causing massive disruptions in the lives of both health‐care employees and patients (Iyengar et al. 2020). Globally, there has been a growth in demand for health‐care services. To meet the increasing demand for healthcare, healthcare providers who had no prior experience dealing with such emergency cases were recruited, putting them at a higher risk of getting the virus (Khatatbeh et al. 2021). Coupled with this insufficient experience from some health care givers are the rapidly changing working environment, creation of new infection control regulations, and the need to adjust care delivery have all posed significant obstacles to health care delivery and management (Catania et al. 2021). Nurses who work in such a critical environment risk being infected and subjected to a great deal of stress which may predispose them to psychological disorders (Huang et al. 2020; Lai et al. 2020).

Twenty nurses caring for COVID‐19 patients were chosen for a study at the First Affiliated Hospital of Henan University of Science and Technology in China (Sun et al. 2020). Using phenomenological approaches, the researchers investigated the psychological experiences of carers of COVID‐19 patients. Their findings revealed that nurses activated psychological defence mechanisms, such as speculation, isolation, distraction, self‐consciousness, humour, and rationalisation as coping strategies to the high psychological demands they faced during care for the patients. COVID‐19 has major repercussions for nurses due to the greater degree of exposure. Sun et al. (2020) reiterated longer job hours, less time with family, and working in a high‐risk workplace were all common stresses for nurses. Another qualitative study by Adatara et al. (2023) in the Volta region of Ghana used a case study design. They purposefully sampled nurses who provided care for COVID‐19 patients, and the nurses reported inadequate training about COVID‐19 and management, logistical challenges, stress, and stigmatisation from friends and families (Adatara et al. 2023). A systematic review by Xu et al. (2021) on the psychosocial experiences of frontline workers revealed that nurses had several mental and emotional reactions to COVID‐19. These reactions were mainly fears of getting infected and possibly transmitting to family members, friends, and work colleagues. Their experiences were further compounded by lack of resources and the physical and professional challenges of using PPEs as they hindered the adequate delivery of nursing care to patients. Exploring the experiences of nurses during the pandemic is important because they are the healthcare professionals who spend most hours with patients. An understanding of their experiences is relevant in illuminating gaps in healthcare systems such as lack of support, inadequate resources, and infrastructure. In addition to that, data from nurses' experiences could guide policymakers in creating more effective emergency response plans, address concerns and needs of nurses, increase job satisfaction and retention since there were accounts of a significant number of nurses who planned to leave or left the profession during and post‐pandemic (de Cordova et al. 2022; Martin et al. 2023).

The COVID‐19 viral pandemic has changed the way health‐care systems interact with patients, employees, and the general public. Many systems have had to adjust or adapt their care processes in response to the pandemic in order to limit the spread of COVID‐19 (Altman et al. 2021). Nurses play a vital role in supporting and assisting COVID‐19 patients in their rehabilitation. This pandemic, in particular, has impacted nurses significantly and poses certain issues for this critical sector of society. It appears that reducing the risk of health workers contracting the virus to zero is very difficult. This difficulty in risk reduction further places patients and other health care workers at risk. This was evidenced in the findings of Vandyck‐Sey et al. (2020) who revealed that nurses and doctors form the majority of the health worker infections in Ghana. Coupled with the setting up of isolation centers, there was the need for a health service special team to be created to work in these isolation centers in many health facilities in the country. This led to a reduction in the number of nurses in the various general wards due to staff deficiency (Yeboah et al. 2024). Delivery of nursing care during the pandemic was highly altered, and examining the experiences of nurses during the pandemic will aid in the modification and improvement of care in the face of any respiratory pandemics in the future. Several studies employing different designs such as case‐study, cross‐sectional, and explorative qualitative approaches have been conducted across different regions to examine nurses' experiences and challenges during care (Adatara et al. 2023), resilience and burnout among nurses (Konlan et al. 2022; Nimako 2021), and provision of antenatal care during COVID‐19 (Acquah‐Mensah and Owusu‐Mensah Richard 2024). The main objective was to explore the general experiences of nurses caring for COVID‐19 patients at two isolation centers in the Ashanti region of Ghana. This objective used a different methodology to validate existing findings and to examine whether previous findings hold true for different contexts.

## 3 Methods

### 3.1 Design

A descriptive phenomenological design was used in this study. We adopted Colaizzi's approach for carrying out phenomenological studies (Morrow et al. 2015).

### 3.2 Setting

The study was conducted among nurses who worked in the COVID‐19 isolation centers of the Kumasi South Hospital and the Manhyia District Hospital, Kumasi. These hospitals were chosen because they are among the large hospitals in the Kumasi Metropolis that received a significant number of cases during the pandemic.

Kumasi South Hospital is the second largest hospital in the southern part of the Ashanti region of Ghana. It is a government regional hospital located specifically in Ashanti, Atonsu‐Agogo, Ghana. The hospital was constructed in 1976, as an urban health center and was later changed to be the Kumasi South Hospital. It was upgraded to the status of Ashanti Regional Hospital in 2002. Manhyia District hospital is located in Manhyia, Kumasi. For years now, the Manhyia District Hospital, Kumasi in Ashanti Region has seen a significant standardisation in terms of quality services, infrastructure, staffs, management among others. It was recognised as the best hospital in 2015, the second best in 2016, and the best in the 2017 after the peer review in the region.

### 3.3 Sampling and Sample Size

The exact number of participants was determined by the number of staff present and data saturation. Initially, all nurses who met the inclusion criteria were considered for recruitment. However, the final number of participants was influenced by contextual constraints, including staff transfers to other hospitals, and some eligible nurses being on leave or otherwise unavailable during the data collection period. Saturation was considered to have been achieved when successive interviews yielded no new themes, insights, or variations in responses relevant to the research questions. Also, participants consistently shared similar perspectives, and all questions in the interview guide were addressed comprehensively without the emergence of any novel information. This redundancy in data was a signal that further data collection was unlikely to contribute additional meaningful insights. A total number of 9 nurses who satisfied the inclusion criteria were involved in the study; 6 nurses from the Kumasi South Hospital and 3 nurses from the Manhyia District hospital. A purposive sampling method (Zhang et al. 2020) was adopted to enrol nurses who worked in the isolation units of the two hospitals. The inclusion criteria included (1) nurses stationed to work in isolation centers who have confirmed that they have cared for COVID‐19 patients and (2) nurses who decided to participate in the study voluntarily.

### 3.4 Data Collection and Analysis

Data was collected using a semi‐structured interview guide (see interview guide in Supporting Information). After consent was sought from the ward in‐charge, the nurses were approached personally by the researchers to recruit them for the study. Researchers introduced themselves to participants, provided rationale for the study and further necessary details as presented in the consent form. All questions asked during the introduction were answered appropriately by the researchers. Data collection began on 15th August 2021 and ended on 17th September 2021. JA, a male Bachelor of Science in Nursing (BSN) holder who was a student, was the interviewer for the study. He received prior training from EAB, who is an expert in qualitative research and nurse scientist for over 15 years. The interviews were conducted in the English language, audio recordings were taken with the phone of the interviewer and transcribed manually. Each interview lasted for about 10 to 15 min. Field notes were taken by KAO and EOI during the interviews. At the Kumasi South Hospital, the interviews were conducted at the COVID‐19 workstation which was designated for only nurses caring for COVID‐19 patients. At the Manhyia District Hospital, interviews were conducted in a separate room next to the hospital's conference room. COVID‐19 protocols such as social distancing, wearing of nose masks and the use of hand sanitisers were ensured to reduce the risk of transmission during the interview.

Reflexivity was maintained throughout the study to enhance the credibility of the findings. The interviewer acknowledged his background as a nursing student and remained conscious of how his prior knowledge, assumptions, and professional orientation could influence data collection and interpretation. To minimise bias, the interviewer adhered closely to the interview guide, used probing questions neutrally, and engaged in regular discussions with the research team to reflect on emerging interpretations and ensure that findings were grounded in participants' accounts.

The data was analysed with Colaizzi's method of data analysis (Morrow et al. 2015) in Microsoft Word. The voice recordings obtained from the interviews were transcribed and checked for consistency. Each transcript was read multiple times by 3 members of the research team to achieve immersion in the data. Significant statements directly related to the phenomenon under study were identified and extracted. Meanings were then formulated from these statements, with careful attention to preserving participants' original intent. The formulated meanings were organised into clusters of themes and subthemes through an iterative process of comparison and discussion among team members. Independent coding was initially conducted, followed by consensus meetings to resolve discrepancies and agree on a final coding framework. An exhaustive description of the phenomenon was then developed by integrating all the emergent themes. This was further refined into a concise statement capturing the fundamental structure of the phenomenon. The following steps indicated in Table 1 were implemented in this process.

**TABLE 1:** The stages of data analysis using Colaizzi's descriptive method.

Action	Stage of data analysis
Transcripts were read numerous times and short notes were taken to explore the meanings ascribed to a phenomenon and the feelings experienced	Acquaintance with the data
2 Key expressions that are directly related to a phenomenon were chosen	2 Key expressions that are directly related to a phenomenon were chosen
3 These key expressions were evaluated, and expressions with similar meanings were created.	3 Formulating meanings from significant statements
4 The meanings were organised into categories, themes, and subthemes	4 Organizing the meanings found into themes
5 The outcomes were combined with a diverse range of life experiences	5 Developing an exhaustive description
6 The phenomenon in question's essential conceptual structure was defined	6 Producing the fundamental structure
7 The findings were validated in a second meeting with the participants, during which their experiences were compared to the findings	7 Seeking verification of the fundamental structure

### 3.5 Ethical Consideration

Ethical approval was sought from the Committee on Human Research Publication and Ethics (CHRPE) of Kwame Nkrumah University of Science and Technology (KNUST) School of Medicine and Dentistry. Administrative approval was also obtained from the various study settings. Consent of participants, both verbal and written, was obtained from the participants. Any aspect of the research in which they were to take part was thoroughly explained to them. Every single person participated willingly in the study. The participants were also informed that they could opt out at any time during the interviews.

Data collected from participants, including identifying information, was presented anonymously to maintain confidentiality. The amount of time spent probing into individuals' personal life was kept to a minimum.

### 3.6 Trustworthiness

Lincoln and Guba evaluative criteria were used in ensuring trustworthiness in the study (Lincoln 1985). Credibility was achieved by ensuring that the interviewer had the training experience to conduct the interviews, and researchers gained an understanding of the research context by spending a significant amount of time in the settings. Transferability was ensured by providing detailed descriptions of all methods used in the study such as data collection and analysis processes. Dependability was ensured by maintaining a comprehensive audit trail of all research activities over the course of the study. Theories and member checking of findings were involved in the process of triangulation to achieve confirmability.

## 4 Results

### 4.1 Demographic Data of Participants

The mean (standard deviation) age of the participants was 29 (3) years, and their ages ranged from 24 to 34 years. Six out of the nine participants were females. Participants had worked as professional nurses for a minimum of 2 years and a maximum of 11 years, with an average (SD) of 4 (3) years. There was an equal number (*n* = 4) of staff nurses and senior staff nurses. Participants had worked in the isolation unit between 4 and 15 months, and the majority were married (*n* = 5) (Refer to Table 2).

**TABLE 2:** Characteristics of participants (*n* = 9).

ID	Age (in years)	Sex	Rank	Working experience (in years)	Duration in isolation unit (in months)	Marital staus
N1	34	F	SSN	11	12	Married
N2	30	F	SN	3	10	Married
N3	29	M	SSN	7	5	Married
N4	31	F	SN	3	15	Married
N5	25	M	SN	2	4	Single
N6	24	F	SN	2	4	Single
N7	32	M	SSN	4	12	Married
N8	27	F	SM	2	4	Single
N9	28	F	SSN	4	12	Single

*Note:* F, female; M, male; N, nurse participant; SM, staff midwife; SN, staff nurse; SSN, senior staff nurse.

Three themes and nine subthemes were identified from the data. The themes were Care context, Covid effects and Coping strategies (Summary in Table 3).

**TABLE 3:** Summary of themes and subthemes.

Themes	Subthemes	Participant comments
Care context	Inadequate supply of equipment	*Anytime I had to improvise PPE's, I don't spend much time on patients. I feel bad about but that is the best thing to do. What if we become incapacitated during the process of giving care? Who will be there to care for the patients? So, I think we had to take care of ourselves as well as we can to stay alive and care for our patients*. (N8).
	Lack of support	*We were just recruited, trained and to begin care. There were times we wondered if the facility actually thought about, we the nurses working in the isolation unit*. (N5)
	Poor working conditions	*At first, we were not here, we were sleeping under the NADMO tent. Oh yes. And anytime it rained, we had to hold the tent firmly to ensure that it does not collapse. It rains and you could see water running under the tent*. (N4)
Covid effects	Improved infection prevention and control	*The things we used to do as IPC methods were actually not the right thing. The training opened our eyes to a lot of things and now I can boast of being an expert in proper wearing of the PPE's*. (N5)
	Psychological effects	*I was scared at first before giving care because I heard a lot of doctors and nurses were dying especially those caring for COVID‐19 patients*. (N1)
	Physical effects	*I got exhausted from caring for a patient whose spO2 was declining. I nearly went black out so I left the patient over there and I tried to save my life too*. (N4)
	Socioeconomic effects	*My husband didn't want me to work here. He was angry at first but he is okay now. My friends, I mean nurses who weren't selected will see you and be like, don't come close to me. You are a COVID nurse. They will be isolating themselves but now it is better and normal*. (N1) *For the economic part, it is a fact that every nurse is a business woman now. But because of the increased workload, we don't have time for business anymore*. (N1)
Coping strategies	Shortage of PPE's	*When there are shortages of PPE's, we use the partial PPE's. With the partial PPE's, the cover all covers only the hands, body and parts of the ankle so we use it with the face shield, mask, gloves, caps and boots. The normal PPE's cover from head to the toes but we always had gloves and the nose masks*. (N1)
	High psychological demands	*We coped with the fear and anxiety as a team. We encouraged ourselves because we understood each other*. (N9) *There were times I isolated myself because I needed to think through these whole changes. It helps me anytime I have any form of fear inside of me*. (N6)

### 4.2 Care Context

The environmental conditions in which the nurses provided care for the isolated patients with COVID‐19 described this theme. Three subthemes emerged from this theme. The participants' statements about the care setting revealed an insufficient supply of equipment, a lack of support, and poor working conditions. Patients' perceptions of the quality of nursing care are influenced by a variety of factors. Nursing is about more than simply the medical side of things. The care environment encompasses a variety of aspects such as equipment availability, support, acceptable working circumstances, and appropriate policies to improve the quality of care provided to patients.

#### 4.2.1 Insufficient Supply of Equipment

According to the participants, basic health care and medical facilities were adequate for providing care to the COVID‐19 patients, but not sufficient. In circumstances where personal protective equipment (PPEs) was sufficient, patients were cared for without interruption since the caregivers were in a highly protected state at the time.

When I am approaching a patient and I know I have all of my required PPE's on, I feel confident because I know I am practicing appropriate barrier nursing care. When it is not available as needed, I try to limit some of my activities around the patient. (N2)

Anytime I had to improvise PPE's, I don't spend much time on patients. I feel bad about it but that is the best thing to do. What if we become incapacitated during the process of giving care? Who will be there to care for the patients? So, I think we had to take care of ourselves as well as we can to stay alive and care for our patients. (N8)

#### 4.2.2 Lack of Support From Authorities

The participants emphasised that support from authorities could aid in the delivery of treatment. When they first started to care, they saw some level of support from the authorities but was unavailable after some time. Since it was a novel strain of virus that they had never dealt with before, they expected a lot more from the authorities.

We were all afraid of the virus, to be honest. So barely seeing and getting updates from the higher authorities was because probably they were afraid of the virus to even come near the isolation unit. (N3)

We did not necessarily need money from the authorities but at least the frequent presence of our leaders and giving us updates on how they are putting things in place for us. (N4)

#### 4.2.3 Poor Working Conditions

Working environments are important to promote employee efficiency even when they are not related to health care. The health‐care industry is no exception. Working conditions, according to the nurses, were appalling. Hospital policies, a change in working units, a deteriorating isolation unit, difficulty collaborating with other team members, and a high risk of infection owing to frequent interaction with patients were among the conditions.

At first, we were not here, we were sleeping under the NADMO tent. Oh yes. And anytime it rained, we had to hold the tent firmly to ensure that it does not collapse. It rains and you could see water running under the tent. (N4)

Some of the doctors were even scared than we were. They come to the unit and choose not to get into contact with the patient. They want us to rather risk our health, go in there and bring the information about the patient so that they can give orders to continue care. (N6)

### 4.3 Covid Effects

This theme was defined as the possible outcomes the pandemic had on nursing care delivery. The effects of the pandemic had varying effects spanning physical, psychological, socioeconomic aspects as well as improving infection prevention and control (IPC) methods.

#### 4.3.1 Improved Infection Prevention and Control

The pandemic also helped the health sector to some extent. Beside it crippling the health sector, it did it some good. The problem is, it did more harm than good. The pandemic paved the way for extensive education and training in the health sector. There was the need for education and training to ensure that health care providers provided the best care to infected patients while protecting themselves as well. All nurses revealed that the training that was organised for them did them a great good. They learnt the correct method of wearing PPE's as well as improving upon the Infection Prevention and Control (IPC) methods.

Positively, it has affected us in the sense that at first IPC's were not actually taken seriously, unlike now. (N9)

The things we used to do as IPC methods were actually not the right thing. The training opened our eyes to a lot of things and now I can boast of being an expert in proper wearing of the PPE's. (N5)

The training helped me to know a lot of things. Now it feels like I am advanced than those in the normal wards because there were a lot of things, we learned which isn't taught in the normal ward. (N6)

#### 4.3.2 Psychological Effects

The state of mind of the nurses during the delivery of care was used to describe this subtheme. The quality of care provided is influenced by the psychological state of the health care providers, and quality is enhanced by a healthy mental state. A high‐stress mental state may have a negative impact on nursing care quality. Fear and worry have a substantial impact on the treatment offered to COVID‐19 patients, according to the nurses.

In the beginning, there was a sense of worry and anxiety. Participants were concerned from the moment they were called by their supervisors to join the COVID‐19 team until they started caring for the patients. Fear and worry were amplified when some of the team members became infected.

I was scared at first before giving care because I heard a lot of doctors and nurses were dying especially those caring for COVID‐19 patients. (N1)

It wasn't easy taking it. I was the first nurse Director called. I didn't like the experience. (N4)

I was scared because we have heard a lot of information about COVID‐19 and how it was damaging the health sector. (N5)

During care of the patients, it was observed that level of anxiety and fear had reduced. Some participants even came to accept the fact that the isolation unit was safer than the normal ward. They supported that with the fact that all patients at the isolation unit are known to be COVID‐19 patients unlike the other wards where asymptomatic positive cases may be around without the health professionals recognizing it.

I am no longer afraid because I know if I wear my PPE's well and cover myself well, nothing will happen. (N1)

The fear left with time and we now nurse the COVID‐19 patients like any other infectious patients. (N3)

I later realized that this place is a lot safer than other units because we know what is at stake and what we are working with but other facilities, they don't. (N5)

#### 4.3.3 Physical Effects

Excess COVID‐19 exposure puts frontline healthcare workers at a much‐increased risk of infection and mortality. The majority of the comments from the participants included fever, cough, dizziness, and burning skin. These consequences were shown to be widespread among nurses who had worked in the isolation unit for at least 3 months, according to the study.

I got exhausted from caring for a patient whose spO2 was declining. I nearly went black out so I left the patient over there and I tried to save my life too. (N4)

It is very hot inside and really burned our skin and sometimes we felt dizzy working inside the PPE's. (N5)

There were times I began to experience high temperature and cough. I even suspected that it might be manifestations of an infection with the virus. (N7)

#### 4.3.4 Socioeconomic Effects

The pandemic had its significant impact on the socioeconomic aspect of the lives of nurses. The social state of an individual's life impacts health. Participants revealed that they suffered from stigmatisation from family and friends. The stigmatisation reduced after they educated their families on the virus and the need to adhere to protocols to ensure safety. Some willingly isolated themselves from their families anytime they began to show some symptoms that overlapped with some of COVID‐19.

My husband didn't want me to work here. He was angry at first but he is okay now. My friends, I mean nurses who weren't selected will see you and be like, don't come close to me. You are a COVID nurse. They will be isolating themselves but now it is better and normal. (N1)

At first, family and friends did not want to get close because they thought I was the virus itself so it affected our relationship. (N2)

At first, we watched football together but since COVID‐19 came, we couldn't do that anymore. With they even having knowledge that you are caring for COVID‐19 patients makes them keep their distance. Family bonds were really affected. (N3)

Participants also revealed that their economic status did not actually change. The only problem they had was with the authorities because they felt they were being cheated on, in the sense that they were supposed to receive some allowances because they worked longer hours than the normal staff. They worked for 10 to 12 h instead of the usual 6 h. Before they began care, they were assured of some incentives but when they began care, they were not receiving as promised.

My financial status didn't really change. My only problem is that we didn't really receive to full measure the financial promises by the government and facility. (N4)

However, some of the participants also revealed that their businesses were affected and that affected their income significantly. They did not provide details of what their businesses entailed but reported that they could not make time for their businesses because they worked extra hours.

For the economic part, it is a fact that every nurse is a businesswoman now. But because of the increased workload, we don't have time for business anymore. (N1)

We don't have time for the other things we did after closing from work to make extra income. (N2)

### 4.4 Coping Strategies

The way nurses developed certain strategies to overcome certain challenges was used to define this theme. Two subthemes emerged from this subtheme; one dealt with coping with the shortage of personal protective equipment (PPE's) and the other dealt with the high psychological demands.

#### 4.4.1 Shortage of PPE'S

The study revealed that all the participants coped with the shortages of PPEs through improvisation. The study also revealed that, apart from improvising, the nurses also decontaminated some of the PPEs for re‐use. Also, in the absence of the full PPEs, they used partial PPEs.

When there are shortages of PPE's, we use the partial PPE's. With the partial PPE's, the cover all covers only the hands, body and parts of the ankle so we use it with the face shield, mask, gloves, caps and boots. The normal PPE's cover from head to the toes but we always had gloves and the nose masks. (N1)

We washed our N95 with chlorine for reuse. (N4)

We improvised, decontaminated and reused PPEs. (N8)

#### 4.4.2 High Psychological Demands

Isolation, normalisation, and teamwork and support were all psychological defence mechanisms activated by all of the nurse participants. This defence mechanism was activated, which facilitated the adaptation to changes.

The COVID‐19 team is very strong. Through everything, we knew we had each other and that was enough to help us through our states of anxiety. (N4)

We realized COVID‐19 is also a virus. The big deal was what we were facing in the isolation unit and not outside. We chose to avoid the negative comments and news and focus on ourselves and patients. That magic helped us. (N7)

We coped with the fear and anxiety as a team. We encouraged ourselves because we understood eachother. (N9)

## 5 Discussion

The study aimed to explore the lived experiences of nurses caring for COVID‐19 patients. The majority of the participants were females; most participants had worked as nurses for less than 5 years. Considering both gender and working experiences of participants, we did not identify any differentiating aspects of their initial and later responses as frontline healthcare workers for COVID‐19 patients. The central theme of their experiences was the fear of contracting the disease and infecting their family members, which was consistent in all participants' responses. A cross‐sectional study by Moradi and Sharififar (2022) reported that female nurses experienced more fear than male nurses, and nurses who had worked for more than 10 years experienced more fear during care for COVID‐19 patients. Our study did not identify these differential levels of fear; however, it is worth noting that different sociodemographic characteristics could influence a healthcare worker's reactions in the face of a pandemic (Liang et al. 2024; Ricalde‐Castillo et al. 2023). In addition to that, the qualitative nature of this study inherently limits the ability to make statistical inference of how there may be differences in the experiences of nurses based on sociodemographic characteristics such as age, marital status, and years of experience. Since most studies exploring nurses' experiences in Ghana have largely been qualitative, future studies could focus on adopting quantitative and mixed methods designs to broaden our current understanding of the reactions and experiences of nurses during the pandemic.

Discomfort caused by the outbreak, strenuous work, and lack of protective materials caused great physical exhaustion among nurses caring for COVID‐19 patients and emerged in this current study. Nurses' concerns regarding friends and family members in this study were similar to those in Adatara et al. (2023), Ahmadidarrehsima et al. (2022), and Muz and Erdoğan Yüce (2021). The study revealed that the threat of the pandemic, physical tiredness, health threats, and insufficient understanding resulted in a huge number of unpleasant emotions such as fear and anxiety. Education of health professionals on infection prevention and control, together with the provision of safe working environments, has the potential to improve healthcare delivery and patient health outcomes. It has been reported that the authorities disseminating information on personal protective equipment, establishing maximum working hours, and suitable shift times to safeguard nurses from overworking, and effective communication could help improve personal and team performance (Adams and Walls 2020).

According to the study, the nurses lacked early psychological counselling. It is best to do a stress assessment and screening of nurses as soon as they receive the pandemic prevention tasks, as well as to give skilled, adaptable, and ongoing psychological support to encourage emotional release and improve the mental health of nurses. These have also been suggested by Smith et al. (2017), Lee et al. (2005), Chen et al. (2021), Fowler and Wholeben (2020), Eslami Akbar et al. (2015) and Spoorthy et al. (2020). There is the need for early support systems, such as adequate supplies of protective materials and a reasonable allocation of human resources. Providing emergency medical supplies is critical to national public health emergency response systems in the event of a pandemic. Our findings imply that health‐care workers are at risk for a variety of health problems as a result of the COVID‐19 pandemic. Fever and cough were the most common symptoms among those infected with COVID‐19. The study revealed that frontline nurses work in close proximity to patients for longer periods of time, and that can lead to exhaustion, stress, and anxiety. This outcome was consistent with the study by Shaukat et al. (2020).

Almost every government in the world has taken steps to prevent COVID‐19 from spreading. These procedures include infection control interventions, quarantines, social distancing requirements, and meeting limitations. While they have helped to lower the pandemic's death and morbidity, they have also contributed to social isolation and stigma (Xiang et al. 2020). Our findings disclosed that nurses faced a high incidence of stigmatisation and altered interpersonal relationships with their loved ones. This is consistent with the study by Sun et al. (2020) who also reported that nurses felt alienated and lonely as a result of their relatives and friends isolating themselves from them for fear of getting the virus. Some parts of society stigmatised the nurses because they thought nurses had the ability to spread the virus. Similarly, during the Middle East Respiratory Syndrome Coronavirus (MERS‐CoV) 2003 epidemic, medical personnel and their families were stigmatised and shunned by society as potential carriers (Kim 2018).

These experiences have been shown to be consistent among nurses in different countries across the globe. Nurses in high income countries such as the United Kingdom, United States of America, and Australia faced as many challenges as nurses in low income countries (Bennett et al. 2020; Coşkun Şimşek and Günay 2021; Jackson et al. 2023; Xu et al. 2021). These similarities in experiences do not necessarily equate to similar healthcare structures and/or responses between high income and low income countries. Firstly, the novel nature of the COVID‐19, coupled with the challenges scientists faced in producing vaccines, the rate of spread and mortality, and the significant changes in workflow at the hospitals all contributed to an increase in global fear and worry (Filip et al. 2022; Goh et al. 2021; Hidaka et al. 2021; Sethi et al. 2020). Secondly, majority of COVID‐19 recorded cases were in the high income countries compared to low income countries like Sub Saharan African countries (Statista 2023). However, the skewed distribution of cases across the globe still exposed the worldwide healthcare systemic weaknesses, and this reflected in the similarities in nurses' experiences across the globe. Amidst the plentiful negative experiences, there were several lessons learnt from the pandemic as reported by participants in the study. Similar feedback has been reported by nurses in other studies and these included improved infection control practices, resilience in managing high psychological demands, enhanced critical thinking skills and decision‐making, and an increased sense of professional commitment (Jackson et al. 2023; Okediran et al. 2020; Sun et al. 2020). These positive experiences made nurses hopeful again and increased confidence their confidence in how to face any future pandemics. These experiences of nurses are important because they contribute to existing knowledge of nurses' pandemic preparedness response. This may necessitate the development of nursing models for pandemic responses. Several models have been adopted to study nursing care during pandemics such as Donabedian's Quality model, Quality Health Outcomes model, Systems Research Organizing model, and System Engineering Initiative for Patient Safety 2.0 model (Bethel et al. 2022). However, these models are adapted from other disciplines and have their limitations in terms of applicability to nursing research and practice. Development of model(s) that originate from nursing may be useful in guiding nursing research and practice in future pandemics.

The COVID‐19 pandemic has left a significant signature in the healthcare system of the world; however, it has also left important lessons such as the importance of multidisciplinary collaboration in pandemic response and opportunities to strengthen the healthcare systems. These lessons are important for all healthcare professionals and would improve the global preparedness and response to future respiratory pandemics.

## 6 Limitation

This is a short‐term research project and does not explore how nurses' experiences may influence delivery of care in the future. In order to better understand experiences of the nurses, a longitudinal study of the participants on how lessons learnt from the experience could translate into modifying future practice might be beneficial.

## 7 Conclusion and Recommendations

The findings of this study reveal that nurses working in COVID‐19 wards and care centers are subjected to stressful mental, emotional, and working environments; despite these challenges, nurses develop coping strategies in order to continue offering proper care to their patients. Nurses are at risk of developing physical and mental health problems as a result of caring for COVID‐19 patients. There is the need to enhance optimum healthcare delivery to COVID‐19 patients and also make the necessary changes to prepare adequately in the face of future pandemics. This entails investing in robust healthcare infrastructure, increasing the supply of essential medical equipment, and ensuring the availability of PPEs for healthcare workers. Training and upskilling healthcare professionals to handle dynamic situations is important, along with establishing clear communication channels and protocols for emergency response.

## Author Contributions

J.A.: conceptualisation, data curation, formal analysis, writing – original draft. J.K.D.: supervision, methodology, writing – review and editing. E.A.B.: methodology, supervision, validation, writing – review and editing. K.A.O.: conceptualisation, data curation, writing – original draft. E.O.I.: methodology, data curation, investigation. S.E.A.O.: conceptualisation, investigation, data curation. F.A.A.: conceptualisation, formal analysis, writing – review and editing. D.G.: data curation, formal analysis, writing – original draft. A.K.A.: project administration, formal analysis, writing – review and editing.

## Funding

Self‐funded the researchers.

## Conflicts of Interest

The authors declare no conflicts of interest.

## Supporting information


**Data S1:** nop270601‐sup‐0001‐Supinfo1.docx.

## Data Availability

The data that support the findings of this study are available from the corresponding author upon reasonable request.
