# Supplementary material for: Experiences of Nurses Caring for Patients With COVID‐19: A Descriptive Phenomenological Study
Source: Nurs Open. 2026 May 19;13(5):e70601. doi: 10.1002/nop2.70601 (PMC13581070; doi:10.1002/nop2.70601)
Supplement: Supplementary file 1 [file NOP2-13-e70601-s001.docx]

**INTERVIEW GUIDE**

1. Please tell me about yourself.

- Rank
- Years of experience by nurse, in the facility.

2. What motivated you to choose nursing?
3. How do you think COVID-19 has affected nursing care in general?

- Daily routine tasks
- Special duties

4. Training on care for COVID-19 patients.

- Who organized it?
- Where was it organized?
- How beneficial was it?

5. How did you first feel when you were recruited as part of the COVID-19 team?
6. What were your general perceptions before beginning care for the COVID-19 patients?
7. How did you cope with shortage of PPE’s and caring for patients diagnosed with COVID-19?
8. A. Have you ever contracted the COVID-19? If yes, how was the experience?

B. Did any of your members contract the virus? If yes, how was the experience?

9. What physical implications has caring for COVID-19 patients have on you?
10. What psychological problems have you experienced when caring for COVID-19 patients?
11. How has your experience affected your relationship with family and friends, as well as your economic status?
